# Supplementary material for: Methyl Gallate Alleviates Acute Ulcerative Colitis by Modulating Gut Microbiota and Inhibiting TLR4/NF-κB Pathway
Source: Int J Mol Sci. 2022 Nov 14;23(22):14024. doi: 10.3390/ijms232214024 (PMC9697899; doi:10.3390/ijms232214024)
Supplement: Supplementary file 1 [file ijms-23-14024-s001.zip › ijms-1971505-Supplementary Materials.pdf]

Supplementary Materials for

Methyl Gallate Alleviates Acute Ulcerative Colitis by Modu-lating Gut Microbiota and Inhibiting TLR4/NF-κB Pathway

Ping Zhou [1,†], Jia Lai [2,†], Yueyue Li [1], Junzhu Deng [1], Fei Yang [2], Shuo Yang [2], Yuesong Wu [2], Xiaoqin Tang [2], Feihong Huang [2,4], Long Wang [2,4], Xinwu Huang [2,4], Qianqian Huang [2,4], Wenjun Zou [1,*], Jianming Wu [3,2,4*]

Includes: Figures S1, S2 and S3; Tables S1 and S2

Supplementary Figure S1

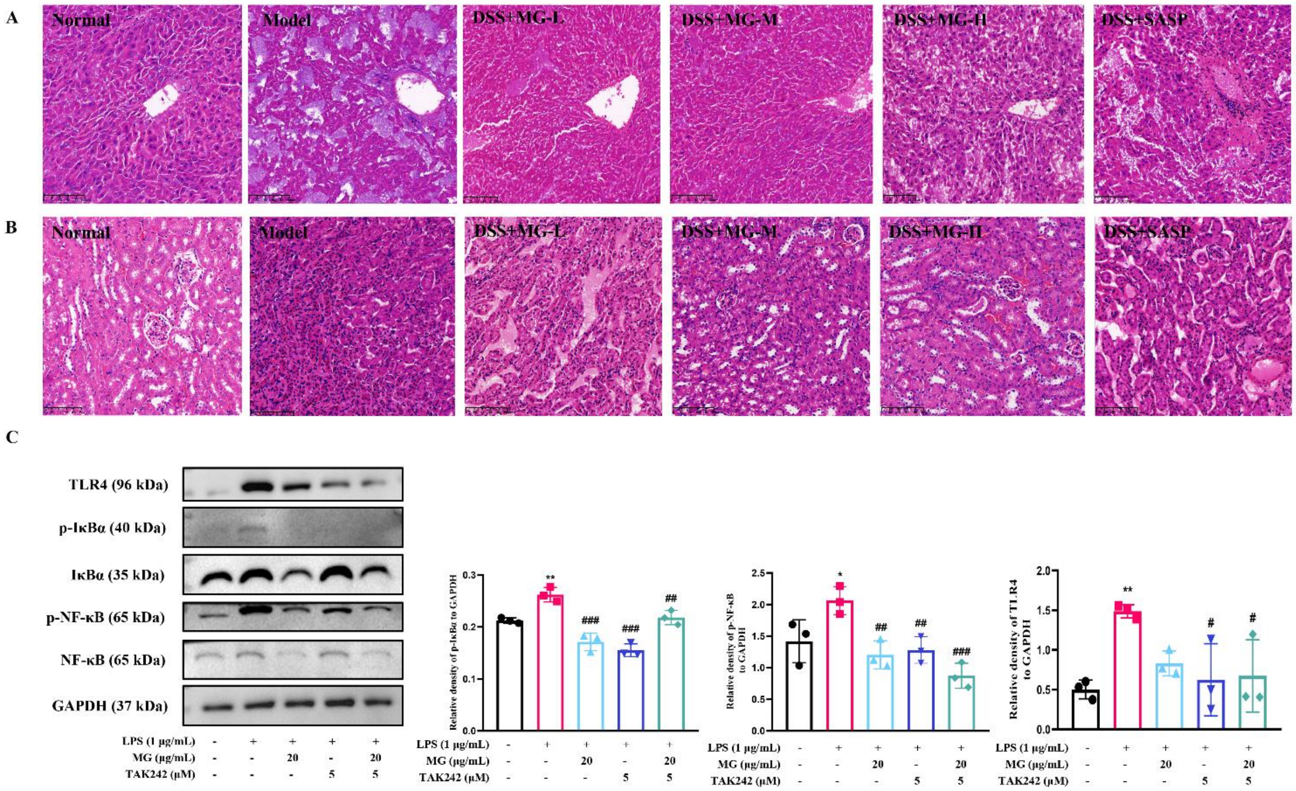

Figure S1. Histopathological observation of colon, liver, and kidney tissues in each group. **(A, B)** Representative sections H&E staining images of liver, and kidney tissue (200×, scale bar: 100 μm). MG and TAK242 inhibited the expression of TLR4/NF-κB signaling pathway in LPS-induced cells. Data were presented as the means ± SEMs of three independent experiments. *p < 0.05, **p < 0.01, and ***p < 0.001 vs the normal group; #p < 0.05, ##p < 0.01, and ###p < 0.001 vs the group treated with only LPS. **(C)**

Supplementary Figure S2

A

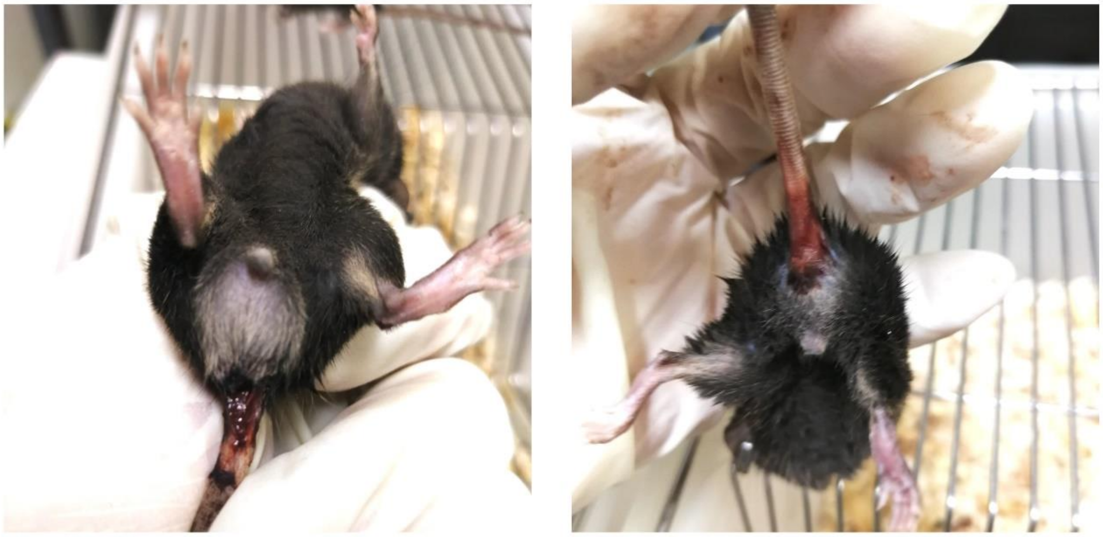

Figure S2. On the eighth day of DSS-induced modeling, hematochezia appeared in the mice. **(A)**

Supplementary Figure S3

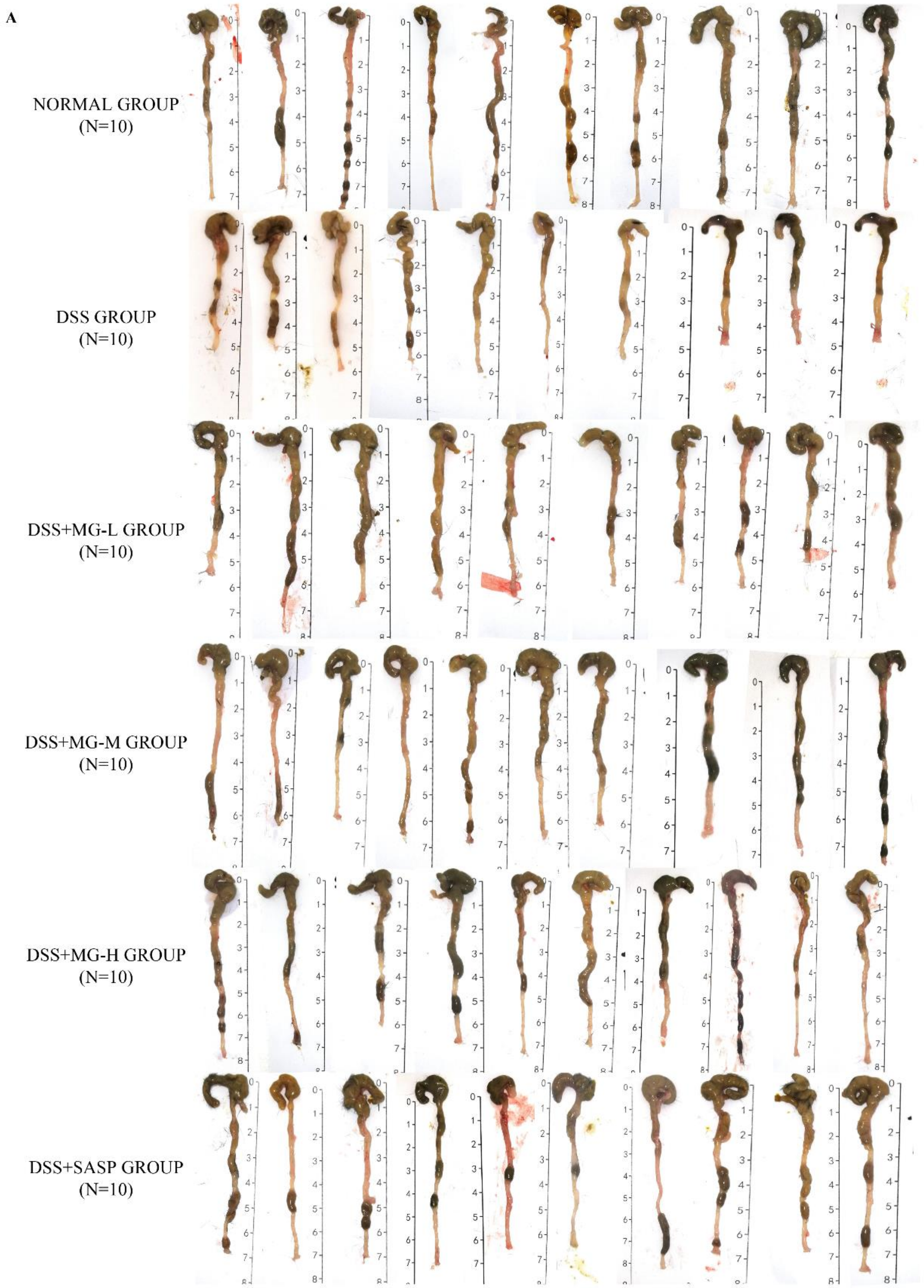

Figure S2. Colonic tissue of mice in each group. **(A)**

Table S1. Disease activity index score.

Score	Weight loss (%)	Stool consistency	Occult blood
0	<0	Normal	Negative
1	1-5	Soft but still formed	+
2	5-10	Loose	++
3	10-20	Very soft and wet	+++
4	＞20	Diarrhea	Gross bleeding

Table S2. Primer sequences used for qPCR amplification.

Gene name	Primer type	Primer sequence
β-actin	Forwad	CATTGCTGACAGGATGCAGAAGG
	Reverse	TGCTGGAAGGTGGACAGTGAGG
TNFα	Forwad	GGTGCCTATGTCTCAGCCTCTT
	Reverse	GCCATAGAACTGATGAGAGGGAG
IL-1β	Forwad	TGGACCTTCCAGGATGAGGACA
	Reverse	GTTCATCTCGGAGCCTGTAGTG
IL6	Forwad	TACCACTTCACAAGTCGGAGGC
	Reverse	CTGCAAGTGCATCATCGTTGTTC
IL4	Forwad	ATCATCGGCATTTTGAACGAGGTC
	Reverse	ACCTTGGAAGCCCTACAGACGA
IL10	Forwad	CGGGAAGACAATAACTGCACCC
	Reverse	CGGTTAGCAGTATGTTGTCCAGC
Arg-1	Forwad	CATTGGCTTGCGAGACGTAGAC
	Reverse	GCTGAAGGTCTCTTCCATCACC
